# Dataset on plot inventories of species diversity and structural parameters of natural and rehabilitated mangrove forest in the Trat province of Thailand

**DOI:** 10.1016/j.dib.2020.105500

**Published:** 2020-04-13

**Authors:** Uday Pimple

**Affiliations:** a The Joint Graduate School of Energy and Environment (JGSEE) and Centre of Excellence on Energy Technology and Environment, King Mongkut's University of Technology Thonburi, Bangkok 10140, Thailand; b Institut Agronomique, Vétérinaire et Forestier de France (Agreenium), AgroParisTech, Paris, France; c CIRAD, UPR Forests, and Societies (F&S), FF-34398 Montpellier, France; d Forests and Societies, University of Montpellier, CIRAD, Montpellier, France

**Keywords:** Rehabilitated mangrove, Species diversity, Mangrove structure, Thailand

## Abstract

The data presented in this article relates to the research article “A history of the rehabilitation of mangroves and an assessment of their diversity and structure using Landsat annual composites (1987–2019) and transect plot inventories” [1]. This article describes how the rehabilitated mangroves evolved over 28 years and whether the ecological parameters of rehabilitated forest resembled those of the adjacent natural mangroves. This article presents species and structural composition data of rehabilitated and adjacent natural mangrove forests in the Trat Province of eastern Thailand. The species type, their girth at 1.3 m breast height, tree height, and the number of seedlings for each of the thirteen species, (*Avicennia alba, Bruguiera cylindrica, Bruguiera gymnorrhiza, Bruguiera sexangula, Ceriops tagal, Excoecaria agallocha, Intsia bijuga, Lumnitzera littorea, Lumnitzera racemosa, Rhizophora apiculata, Rhizophora mucronata, Xylocarpus granatum,* and *Xylocarpus moluccensis*), of the mangrove forests were collected. The data were collected in 10 × 10 m size plots from the seaward to landward end along with the stand age of the rehabilitated mangroves. The data was analysed using the Importance Value, Complexity Index, Simpson's Dominance Index of diversity and Simpson's Reciprocal, and Shannon-Weaver Index to distinguish various diversity and structural parameters using the mangrove forest structure and vegan: Community Ecology Package in R programming [2,3].

Specifications table

**Table:** 

Subject	Biological sciences
Specific subject area	*Forestry, Forest ecology and management, Restoration*
Type of data	Excel file
How data were acquired	Species diversity and structural parameters were obtained using the systematic sampling design (fixed-area sampling), across the mangrove forest landscape. Procedure of data sampling described in Sampling design, paragraph 2.2 in Data and methods of Pimple et al. [1].
Data format	Raw, analysed
Parameters for data collection	Mangrove species richness and structural characteristic variables were aggregated at the plot level. The following dendrometric parameters were measured from each tree species of each plot: forest type, tree diameter at breast height, tree height, crown cover, and the number of seedlings
Description of data collection	The systematic sampling design (fixed-area sampling) across the mangrove forest landscape. A transect line was systematically established perpendicular to the coastline (i.e. With seaward and landward limits), recording the species distributions along the intertidal zones
Data source location	The transect line (from 239,249 m E, 1,350,069 m N to 240,518 m E,1,352,337 m N (UTM Zone 48 datum)) was 2.67 km in length, from the shoreline (seaward end) to the end of the forest (landward end).
Data accessibility	URL: http://dx.doi.org/10.17632/tdbm9dgw9r.1
Related research article	Pimple, U., Simonetti, D., Hinks, I., Oszwald, J., Berger, U., Pungkul, S., Leadprathom, K., Pravinvongvuthi, T., Maprasoap, P., Gond, V. (2020). A history of the rehabilitation of mangroves and an assessment of their diversity and structure using Landsat annual composites (1987–2019) and transect plot inventories. Forest Ecology and Management, 462, 118,007. https://doi.org/10.1016/j.foreco.2020.118007

**Value of the data**

• The species richness, structural parameters, number of seedlings per plot and stand age, between rehabilitated and adjacent natural mangrove stand is very important to assess the degree of success or failure of rehabilitation management practices in the region.
• The data presented here is crucial for understanding the evolution, and potential changes in future ecological variables of rehabilitated mangrove ecosystem.
• The data can be used to compare the structure and species communities in other mangrove forests.
• The data can be used to fit site and species specific above ground biomass models and conversion factors which are very important to estimate the carbon sequestration potential of rehabilitated mangroves compared with adjacent natural stands.
• The data can be used to validate the satellite based species and height predictions across the intertidal zone.

## 1 Data description

The data presented here is the original data that relates to the research article “A history of the rehabilitation of mangroves and an assessment of their diversity and structure using Landsat annual composites (1987–2019) and transect plot inventories” [1]. The data is presented as a single .xlsx file, with the title “DataTrat2019”. The data sheet contains parameters of indigenous and rehabilitated mangrove forest that includes plot number, province (location), forest type, species, number of trees in each plot, abbreviations (as used in article [1] for the name of the species), branches, mean girth at breast height, mean height per species, number of seedlings per plot, and age (only applicable for rehabilitated mangroves).

## 2 Experimental design, materials, and methods

A transect line was systematically established perpendicular to the coastline (i.e. with seaward and landward limits), covering the species distributions along the intertidal zones. In total, 24 plots (10 × 10 m), were established with 100 m apart. Each plot was used to determine the stand composition and diversity, and distributions among the intertidal zones. Field inventories were performed in December 2019. The data were analysed and interpreted. Plots 1 to 15 were in the adjacent natural zone, while plots 16 to 24 were in the rehabilitated mangrove zone. The number of seedlings per plot were counted manually. The plantation dates of the rehabilitated mangroves were obtained from the Trat provincial office of Department of Marine and Coastal Resources, Thailand. The species in each plot were compared to determine the species diversity and structural variations between the rehabilitated and adjacent natural mangroves. The data was interpreted using diversity and structural indices (Importance value, Complexity index, Simpson's dominance index of diversity and Simpson's reciprocal, and Shannon-Weaver index) to derive species diversity, dominance and structural variability (described in [2,3]).
